# Evaluation of GDH and HDP as novel serological biomarkers for *Plasmodium falciparum* malaria diagnosis across varying parasitemia and transmission settings in India

**DOI:** 10.1371/journal.pone.0334313

**Published:** 2025-10-15

**Authors:** Amreen Ahmad, Shrikant Nema, Kushagri Arora, Ruhi Sikka, Akansha Singh, Sri Krishna, Mrigendra Pal Singh, Anil Kumar Verma, Nitika Nitika, Anup R. Anvikar, Himanshu Gupta, Praveen Kumar Bharti

**Affiliations:** 1 ICMR-National Institute of Research in Tribal Health, Jabalpur, Madhya Pradesh, India; 2 ICMR-National Institute of Malaria Research, Dwarka, Delhi, India; 3 Department of Biotechnology, Institute of Applied Sciences and Humanities, GLA University, Mathura, India; Addis Ababa University, ETHIOPIA

## Abstract

Malaria diagnosis has progressed significantly with the introduction of Rapid Diagnostic Tests (RDTs) targeting Histidine-Rich Protein 2 (HRP2), Lactate dehydrogenase (LDH), and aldolase. However, challenges remain, such as the presence of *Plasmodium falciparum histidine-rich protein 2 (pfhrp2)* gene deletions and reduced sensitivity in regions with low parasitemia. This study evaluates antibody responses to novel biomarkers—glutamate dehydrogenase (GDH) and Heam detoxification protein (HDP)—in comparison to conventional antigens (HRP2, LDH, and aldolase) across different infection, transmission settings, and parasitemia conditions. We analyzed plasma samples from 928 participants, including 886 *P. falciparum*-positive cases, using enzyme-linked immunosorbent assay. Our results revealed antibody levels against all studied peptides clear differentiation between malaria-positive and negative samples, with Receiver Operating Characteristic (ROC) curve analysis showing high diagnostic accuracy (Area under the ROC curve > 96%) for detecting *P. falciparum* infection. However, the sensitivity and specificity for differentiating between endemicity or parasitemia groups were limited for certain peptides. Specifically, GDH2 and HRP2 effectively distinguished parasitemia levels, while GDH2, HDP2, and LDH showed promise in distinguishing between varying endemicity levels. These findings suggest that GDH and HDP have significant potential as reliable serological biomarkers for malaria detection. However, further studies are needed to refine their application in categorizing endemicity and parasitemia. This research highlights the need for adaptable diagnostic tools to address the complex challenges of malaria in endemic regions.

## Introduction

Malaria remains a significant global health challenge, especially in tropical and subtropical regions. In 2022, India accounted for 66% of South-east Asia’s malaria cases, with over half caused by *Plasmodium falciparum* [1], and approximately 2 million cases were estimated in 2023 [2]. Effective control and elimination strategies rely on accurate diagnostics; however, the limitations of existing methods, particularly in resource-limited settings, hinder these efforts [3,4]. While in microscopy, Giemsa-stained blood smear remains the ‘gold standard’ method [5], Rapid Diagnostic Tests (RDTs) based on *P. falciparum* histidine-rich protein 2 (PfHRP2) antigen are widely used due to their simplicity. However, *pfhrp2* gene deletion and antigen persistence post-infection undermine RDT accuracy [6], complicating *P. falciparum* malaria detection. The World Health Organization (WHO) recommends using non-HRP2-based RDTs, such as those detecting *P. falciparum* lactate dehydrogenase (PfLDH) or aldolase, in regions with reported 5% *pfhrp2* deletions [7]. Aldolase-based tests can be used in combination with PfHRP2 for detecting *falciparum* and non-*falciparum* species, while PfLDH and Pan-*Plasmodium* (pLDH) based tests are widely used to detect *P. falciparum* and other *Plasmodium* species [8]. However, these tests have lower sensitivity and specificity [9] and are less effective at detecting low parasite levels compared to HRP2-based RDTs [10]. Thus, identifying new diagnostic targets is crucial to address these limitations.

Given the limitations of current RDTs, there is a pressing need to identify novel, reliable diagnostic markers for *P. falciparum*. In this context, glutamate dehydrogenase (GDH) and Heam detoxification protein (HDP) have emerged as promising candidates based on their roles in parasite biology and stage-specific expression for *P. falciparum* diagnosis [11]. GDH plays a key role in glutamate’s catabolism and ammonium’s assimilation, serving as the primary NAD(P)+ source when parasites invade erythrocytes [12]. Importantly, GDH is exclusively expressed in the parasite’s erythrocytic stage and localized in the cytosol [13]. *P. falciparum* has three *gdh* genes, which encode the following proteins: pGDH1 and pGDH2 on chromosome 14, and pGDH3 on chromosome 8. HDP, which is involved in hemoglobin degradation and neutralizing heme toxicity essential for the parasite’s survival [14,15], is encoded by a single gene expressed during the erythrocytic stages. It shows limited genetic diversity among Indian and African isolates and is highly conserved across *Plasmodium* species [11].

This study compares immune responses to GDH and HDP with current RDT antigens (HRP2, LDH, aldolase). Previous findings indicated strong immune responses to PfGDH [13] and PfHDP [11] among *P. falciparum* patients from a single site. Here, antibody levels against all selected antigens were assessed in malaria patients and compared to malaria-slide negative individuals collected from 12 endemic Indian sites. Antibody levels were also compared among patients with parasite densities ranging from ≤200 (PD1), > 200 to <1000 (PD2), and >1000 (PD3) parasites/µL. Additionally, levels were compared across regions with high, moderate, and low transmission intensities. These findings aim to improve diagnostic strategies.

## Materials and methods

### Study site and sample collection

**Fig 1:**
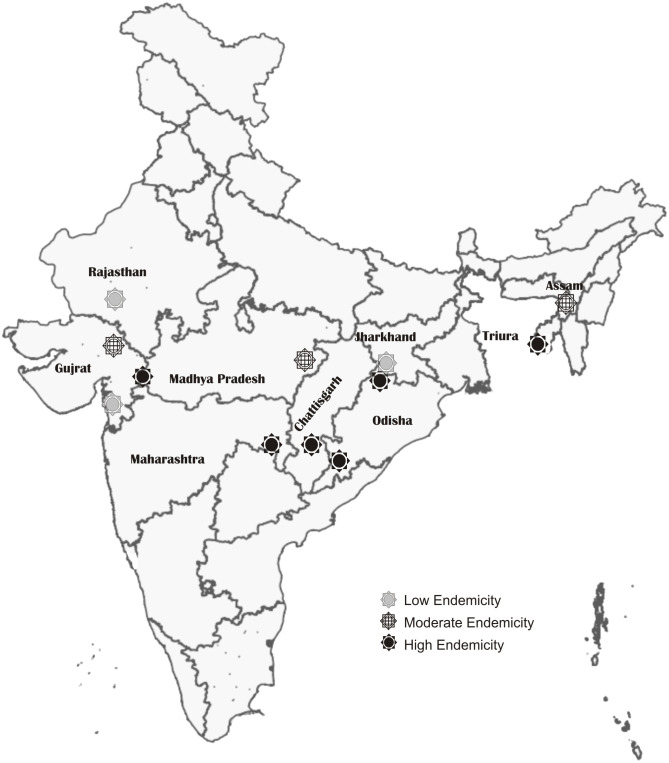
Map showing the study sites from nine malaria-endemic states of India (Source: Survey of India).

Plasma samples (n = 928) were obtained from a previous study [16] conducted across 12 sites in nine malaria-endemic states of India (Fig 1). In brief, microscopically positive *P. falciparum* samples, along with the malaria-slide negative controls, were collected from July to December 2014 across multiple sites during the rainy and peak transmission season. The study covered Odisha, Chhattisgarh, Jharkhand, Madhya Pradesh, Maharashtra, Rajasthan, Gujarat, Assam, and Tripura, selecting two community health centers (CHCs) per state representing high and low endemic regions.

Febrile patients (axillary temperature ≥ 37.5°C or fever in the past 48 h) visiting CHCs for malaria diagnosis were screened, and those positive for *P. falciparum* mono-infection (confirmed by microscopy) were enrolled. Blood smears were stained with Jaswant Singh–Bhattacharji [17] stain, examined by two independent microscopists, and discrepancies were resolved by a third expert. Parasite densities were calculated using standard methods (Parasites/μL = no. of asexual parasites X 8000/no. of WBC counted) [18]. Venous blood samples (1–2 mL) were collected, plasma separated, and stored under freezing conditions for molecular studies. Treatment followed the NVBDCP guidelines, and pregnant women were excluded from the study. Based on the annual parasite incidence (API), these sites were classified as high (API > 5), moderate (API 2–5), and low (API < 2) transmission settings during the sample collection.

### Ethical considerations

Ethical clearance was obtained from the Institutional Ethics Committee of Indian Council of Medical Research-National Institute of Research in Tribal Health (ICMR-NIRTH), Jabalpur (RMRCT/IEC/575/2012, dated: 08/05/2012). The approval remained valid for the period of sample collection, and the study was conducted in accordance with the ethical guidelines for biomedical research on human participants of Indian Council of Medical Research (ICMR) published in 2006.

Written informed consent was obtained from all adult participants, and from the parents/guardians in the case of children. The consent form was also provided and explained in Hindi as well as local language, with the help of a local translator when needed. All individuals diagnosed with malaria were treated as per the national guidelines for diagnosis and treatment of malaria 2013 by the National Center for Vector Borne Diseases Control, (NCVBDC, 2013). The copies of the informed consent form and ethics approval letter are provided as supporting documents.

### Identification of linear B cell epitopes and amino acid repeats

Linear B-cell epitopes in PfGDH, PfHDP, PfHRP2, PfLDH, and PfAldolase from the *P. falciparum* 3D7 strain were identified using the Bepipred 2.0 Linear Epitope Prediction software [19]. This tool, integrated with the Immune Epitope Database and Analysis Resource (IEDB-AR), applies parameters such as a center position of 4 and a threshold value of 0.500. Amino acid repeats in the protein sequence of *Pf34* from *P. falciparum* 3D7 (PF3D7_0419700) were identified both manually and using the ProtRepeatsDB- Direp server [20].

### Peptide designing

Peptides were designed to target different regions, as detailed in Table 1. For the three GDH proteins, distinct regions were selected. Two peptides were designed for both HDP and aldolase, targeting the N terminal and mid-regions. An additional alanine was added to the N-terminus of each peptide to improve binding efficiency to ELISA plates. For both HRP2 and LDH peptides, the N terminal regions were selected. The peptide designs were analyzed using synthesis and analysis tools from Thermofisher Scientific. All peptides were commercially synthesized by Sigma-Aldrich with a purity exceeding 95%.

**Table 1:** Sequences of synthetic peptides used in this study.

Synthetic peptide	Peptide sequences	Location
HRP2	ADAHHAHHAAD	N terminal
LDH	AKSDKEWNRDD	N terminal
Aldolase 1	ACTEYMNAPKK	N terminal
Aldolase 2	AEKSTQGLDG	Mid region
GDH1	AMKYGENNMKN	N terminal
GDH2	ANLNNSGEATL	Mid region
GDH3	ANEQYSSDKYFPT	C terminal
HDP 1	ARKPQKVTNDPESINR	N terminal
HDP2	AESFNKLYNDENKLSE	Mid region

### Enzyme-linked immunosorbent assay

Lyophilized synthetic peptides were dissolved in Milli Q water and diluted to a final concentration of 2 µg/mL in a coating buffer containing 50mM sodium carbonate and 50mM sodium bicarbonate (pH 9.6). Maxisorp ELISA plates (NUNC, Denmark) were coated with 200ng of peptide per well and incubated overnight at 4°C. Next morning, the plates were blocked with 200µl of 5% non-fat dry milk (Sigma-Aldrich) in phosphate buffer saline (PBS) for 1 hour at 37°C. After blocking, plates were washed three times with PBS containing 0.1% Tween 20 (PBST). Plasma samples were diluted at a ratio of 1:200 in an assay diluent consisting of PBST with 5% non-fat dry milk. A volume of 100 µl of the diluted plasma samples was added in duplicate and incubated for 1 hour at 37°C.The plates were washed four times in PBST and incubated for 1 hour at 37°C following the addition of horseradish peroxidase-conjugated goat-antihuman-IgG (Genei, Bangalore; 1:4000 in PBS-5% skimmed milk, 0.1% Tweeen-20). The assay was developed by adding the tetra-methylbenzidine enzyme substrate (Genei, Bangalore) and incubated for 30 minutes at 37°C. The reaction was stopped with 2M H_2_SO_4_. The optical density (OD) was measured at 450 nm using an ELISA plate reader [21].

To determine the specificity of the assay, plasma samples from 16 individuals from a non-malaria endemic area (not exposed to malaria) were used as negative controls. Prevalence of total IgG immune response for the different antigens were considered positive if their OD values were higher than the mean plus two standard deviations of the negative control after subtraction [21].

### Statistical analysis

Principal component analysis (PCA) was performed using the R package prcomp, with visualization done via the ggbiplot package. Antibody levels for each peptide were compared between groups using the Mann-Whitney U test. Receiver Operating Characteristic (ROC) curves were used to evaluate the accuracy of antibody levels in detecting *P. falciparum* patients, distinguishing parasitemia levels, and categorizing transmission conditions. The area under the ROC curve (AUC) with 95% confidence intervals (CI), sensitivity, and specificity were calculated to determine the discriminatory power of the studied peptides. A two-sided p < 0.05 was considered statistically significant. All statistical analyses were performed using R 4.3.3 (R Foundation for Statistical Computing) and GraphPad Prism 10 (GraphPad Software, Inc.).

## Results

### Patient characteristics

We conducted an immunological analysis on samples from 928 participants. Among these, 886 tested positives for *P. falciparum*, while 42 were malaria-slide negative. Based on parasite density, 12.6% (n = 112) of the patients had a density of less than 200 parasites/µl (PD1), 42.1% (n = 373) had a density between 200 and 1000 parasites/µl (PD2), and 45.3% (n = 401) had a density exceeding 1000 parasites/µl (PD3). When categorized by transmission intensity, 12.6% of patients (n = 112) were from low-transmission regions, 22.8% (n = 202) from moderate-transmission regions, and 64.6% (n = 572) from high-transmission regions.

### PCA analysis of IgG antibody levels across sample classifications

**Fig 2:**
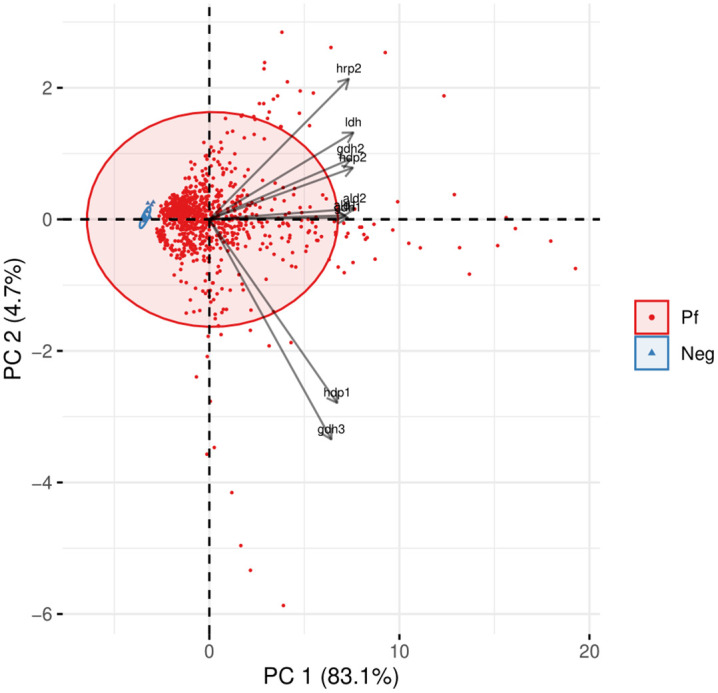
In principal component analysis (PCA), X axis shows principal component 1 and Y axis shows principal component 2 with 83.1% and 4.7% of the total variance, respectively. Antibody levels obtained against nine studied peptides were included in the PCA analysis. Ellipse shapes are showing clustering of *P. falciparum*-positive and malaria-slide negative group samples.

PCA was performed to assess patterns in antibody levels against nine peptides (GDH1, GDH2, GDH3, HDP1, HDP2, ALD1, ALD2, HRP2, and LDH) under three classification methods: infection status (*P. falciparum*-positive vs. malaria-slide negative), three endemicity levels, and varying parasitemia levels. Fig 2 illustrates the PCA plot for the case-control classification, where the first two PCs (PC1 and PC2) accounted for 83.1% and 4.7% of the total variance, respectively. The PCA plot revealed two well-defined clusters, with *P. falciparum* positive samples (red) and malaria-slide negative samples (blue) distinctly separated, indicating robust differentiation based on infection status. S1 and S2 Fig present the PCA results for classifications based on endemicity levels and parasitemia levels, respectively. In both cases, PC1 and PC2 explained similar proportions of the variance (82.1% and 5.0%). However, no distinct clustering patterns were observed for either endemicity or parasitemia levels.

### Differentiation of sample groups using IgG antibody levels

**Fig 3:**
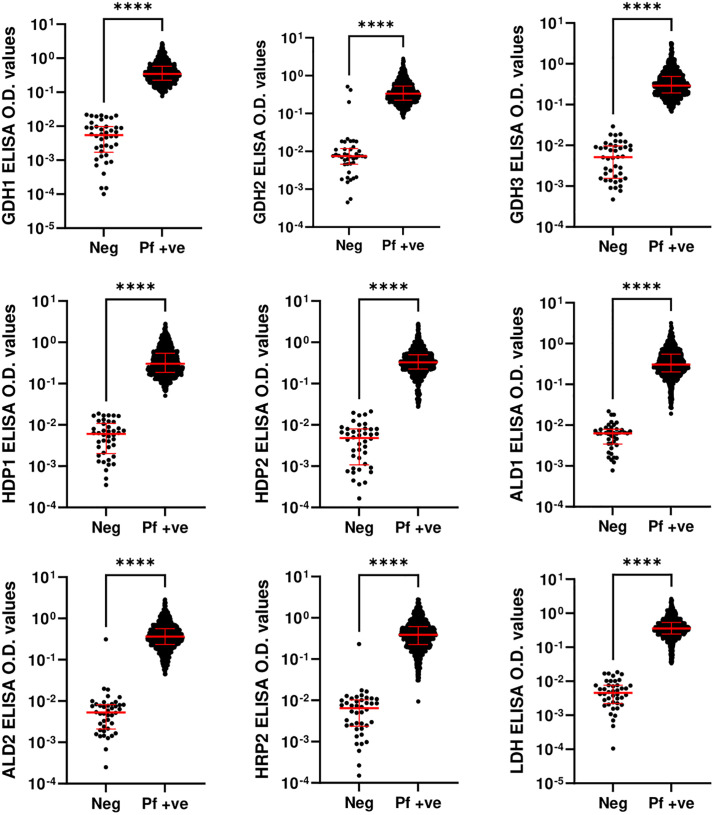
Comparison of antibody levels obtained against nine studied peptides between *P. falciparum*-positive and malaria-slide negative groups. Statistical differences were determined using the Mann-Whitney U test. T bars represent the median and interquartile ranges. (*p < 0.05, **p < 0.005, ***p < 0.0005, ****p < 0.00005).

**Fig 4:**
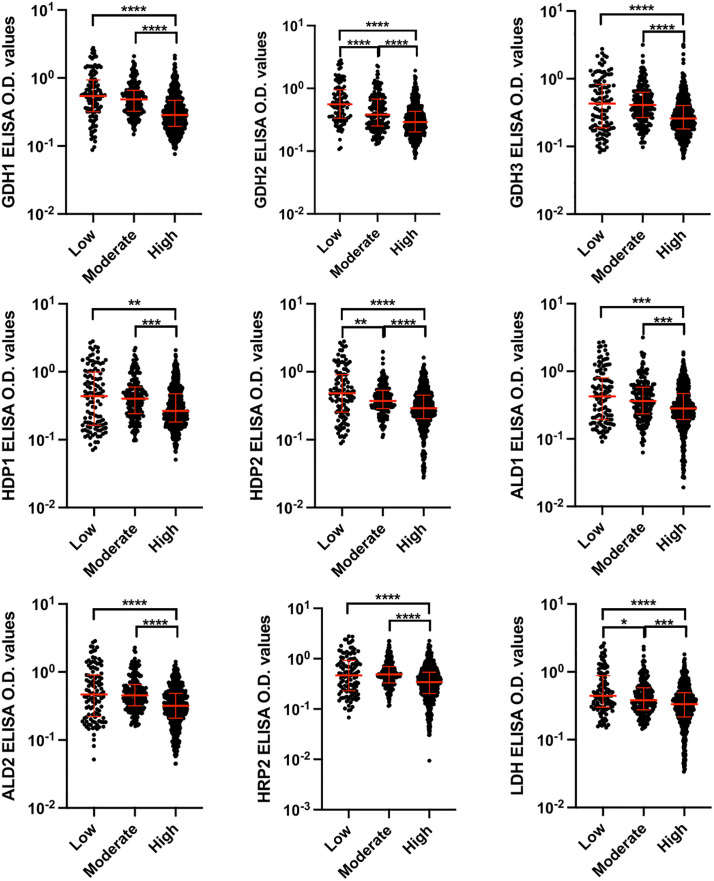
Comparison of antibody levels obtained against nine studied peptides between low, moderate and high endemicity groups. Statistical differences were determined using the Mann-Whitney U test. T bars represent the median and interquartile ranges. (*p < 0.05, **p < 0.005, ***p < 0.0005, ****p < 0.00005).

**Fig 5:**
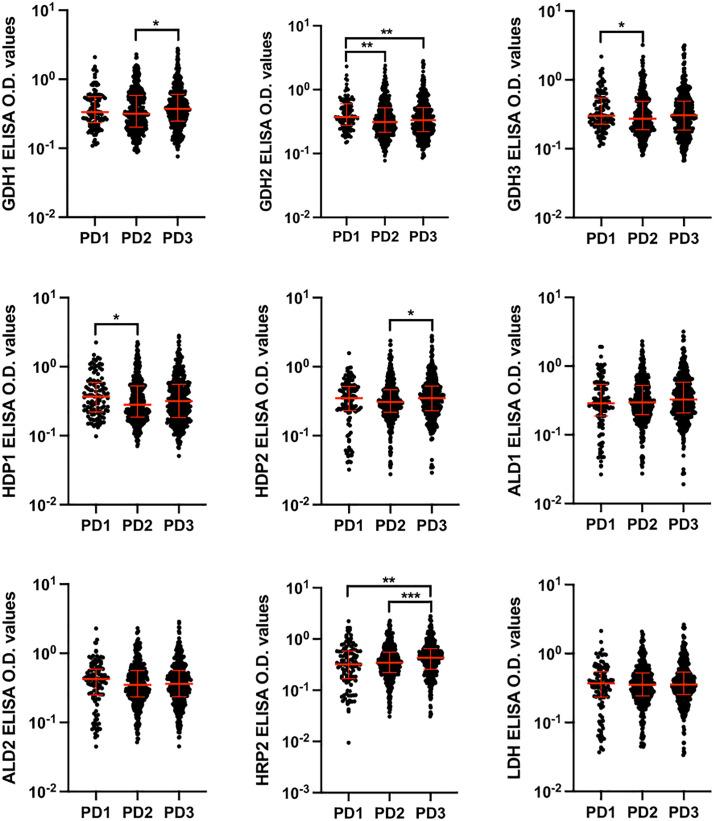
Comparison of antibody levels obtained against nine studied peptides between different parasitemia groups including PD1(<200 parasites), PD2 (>200 to <1000 parasites) and PD3 (>1000 parasites). Statistical differences were determined using the Mann-Whitney U test. T bars represent the median and interquartile ranges. (*p < 0.05, **p < 0.005, ***p < 0.0005, ****p < 0.00005).

Using the Mann-Whitney U test, the antibody levels against nine peptides were assessed to evaluate their ability to distinguish between *P. falciparum*-positive and malaria-slide negative groups, as well as across different endemicity and parasitemia levels. A significant difference in antibody levels was observed between *P. falciparum*-positive (n = 886) and malaria-slide negative (n = 42) groups (Fig 3). When comparing endemicity levels—low (n = 112), moderate (n = 202), and high (n = 572)—antibody levels were significantly different overall. However, responses to six peptides (GDH1, GDH3, HDP1, ALD1, ALD2, and HRP2) did not show significant differences between low and moderate endemicity groups (Fig 4). For parasitemia levels, the samples were grouped into PD1 (n = 112), PD2 (n = 373), and PD3 (n = 401). Antibody levels against GDH1, HDP2 and HRP2 differentiated between PD2 and PD3 (p < 0.05), while GDH2, GDH3 and HDP1 differentiated between PD1 and PD2 (p < 0.05). Additionally, GDH2 and HRP2 differentiated between PD1 and PD3 (p < 0.005). Antibody responses to the remaining peptides did not show significant variation across parasitemia levels (Fig 5).

### Area under the ROC curve analysis of IgG antibody levels

The discriminatory performance of antibody levels against the nine peptides was evaluated using the area under the ROC curve to distinguish between the sample groups. As shown in Table 2, antibody levels against all peptides exhibited excellent performance in distinguishing *P. falciparum-*positive samples from malaria-slide negative samples, with AUC values exceeding 96% and p-values < 0.005. However, the AUC analysis did not perform as well when distinguishing between samples from different endemicity levels (Table 3) or parasitemia levels (Table 4). In both cases, the accuracy remained at or below 75%, with suboptimal sensitivity and specificity.

**Table 2:** Results of the receiver operating characteristic (ROC) curve analysis to detect the *Plasmodium falciparum* patients.

	Markers	AUC (95%CI) [%]	p value	Cutoff	Sensitivity [%]	Specificity [%]
*P. falciparum vs*. Negative	GDH1	100 (100−100)	<0.001	0.02132	100	97.6
	GDH2	96.3 (91.8-100)	<0.001	0.04936	100	92.9
	GDH3	100 (100−100)	<0.001	0.04817	100	100
	HDP1	100 (100−100)	<0.001	0.03477	100	100
	HDP2	100 (100−100)	<0.001	0.02445	100	100
	ALD1	100 (99.9-100)	<0.001	0.02424	99.9	100
	ALD2	99 (97.1-100)	<0.001	0.03240	100	97.6
	HRP2	99.3 (98.2-100)	<0.001	0.02408	99.9	97.6
	LDH	100 (100−100)	<0.001	0.02623	100	100

*AUC: Area under the ROC curve; CI: confidence interval.*

**Table 3:** Results from the receiver operating characteristic (ROC) curve analysis were used to evaluate the performance of different markers under various transmission conditions.

	Markers	AUC (95%CI) [%]	p value	Cutoff	Sensitivity [%]	Specificity [%]
Low *vs*. Moderate	GDH1	54.5 (47.3-61.6)	0.1865	–	–	–
	GDH2	63.3 (56.9-69.7)	<0.001	0.3129	40	80
	GDH3	50.4 (42.9-57.7)	0.9163	–	–	–
	HDP1	50.1 (42.6-57.6)	0.9793	–	–	–
	HDP2	59.1 (51.8-66.4)	0.0074	0.3389	43	68
	ALD1	52.4 (45.2-59.6)	0.4851	–	–	–
	ALD2	50.2 (42.8-57.6)	0.9627	–	–	–
	HRP2	52.1 (44.7-59.5)	0.5450	–	–	–
	LDH	57.3 (50.5-64.1)	0.0316	0.3382	40	68
Low *vs*. High	GDH1	70.7 (65.1-76.4)	<0.001	0.3142	56	78
	GDH2	75.6 (70.1-80.5)	<0.001	0.3146	55	80
	GDH3	63.1 (56.4-69.7)	<0.001	0.2588	50	64
	HDP1	58.4 (51.4-65.4)	0.0048	0.2282	40	62
	HDP2	67.8 (61.7-73.9)	<0.001	0.3153	55	70
	ALD1	60.5 (54.1-66.9)	<0.001	0.2485	40	64
	ALD2	64.4 (58.2-70.7)	<0.001	0.2704	40	70
	HRP2	61.8 (55.7-67.9)	<0.001	0.2775	40	71
	LDH	65.8 (60.3-71.4)	<0.001	0.3345	50	70
Moderate *vs*. High	GDH1	71.6 (67.8-75.3)	<0.001	0.3250	58	75
	GDH2	63.6 (59.1-68.1)	<0.001	0.2528	40	75
	GDH3	68.4 (64.2-72.5)	<0.001	0.2727	53	75
	HDP1	63.1 (58.7-67.5)	<0.001	0.2426	44	75
	HDP2	63.2 (59.0-67.3)	<0.001	0.2888	50	75
	ALD1	60.2 (55.8-64.7)	<0.001	0.2485	40	72
	ALD2	68.3 (64.3-72.3)	<0.001	0.3212	51	75
	HRP2	66.8 (62.8-70.8)	<0.001	0.3321	48	75
	LDH	59.8 (55.5-64.2)	<0.001	0.2865	40	73

*AUC: *Area under the ROC curve; CI: confidence interval. ‘**–‘ *indicates values not shown due to non-significant p value.*

**Table 4:** Results from the receiver operating characteristic (ROC) curve analysis were used to evaluate the performance of different markers under various parasitemia levels.

	Markers	AUC (95%CI) [%]	p value	Cutoff	Sensitivity [%]	Specificity [%]
PD1 *vs*. PD2	GDH1	54.7 (49.2-60.3)	0.1083	–	–	–
	GDH2	59.7 (54.1-65.3)	0.0018	0.3161	51	68
	GDH3	56.8 (50.9-62.5)	0.0300	0.2626	48	61
	HDP1	57.2 (51.4-63.0)	0.0201	0.2756	50	61
	HDP2	50.9 (44.0-57.2)	0.7775	–	–	–
	ALD1	52.7 (46.9-58.6)	0.3516	–	–	–
	ALD2	52.6 (47.2-57.9)	0.3798	–	–	–
	HRP2	53.2 (46.6-59.7)	0.3107	–	–	–
	LDH	53.5 (47.9-59.2)	0.2293	–	–	–
PD1 *vs*. PD3	GDH1	52.0 (46.1-58.0)	0.5062	–	–	–
	GDH2	58.5 (53.0-64.1)	0.0056	0.3314	50	63
	GDH3	54.6 (46.9-62.3)	0.5145	–	–	–
	HDP1	51.2 (43.7-58.7)	0.8610	–	–	–
	HDP2	50.8 (39.4-62.2)	0.9098	–	–	–
	ALD1	50.7 (41.3-60)	0.9233	–	–	–
	ALD2	51.4 (42.5-60.2)	0.8443	–	–	–
	HRP2	59.1 (52.8-65.5)	0.0029	0.3209	68	50
	LDH	55.7 (44.9-66.6)	0.4182	–	–	–
PD2 *vs*. PD3	GDH1	54.9 (50.8-58.9)	0.0185	0.3134	62	50
	GDH2	60.4 (47.8-72.9)	0.1696	–	–	–
	GDH3	52.8 (41.4-64.2)	0.7075	–	–	–
	HDP1	51.6 (40.3-62.7)	0.8373	–	–	–
	HDP2	54.4 (50.3-58.5)	0.0343	0.3505	50	61
	ALD1	51.8 (40.6-62.9)	0.8098	–	–	–
	ALD2	52.9 (40.4-65.5)	0.6970	–	–	–
	HRP2	57.8 (53.1-61.2)	0.0005	0.3451	65	50
	LDH	52.2 (39.0-65.5)	0.7662	–	–	–

*PD1: 1–200 parasites; PD2: 201–1000 parasites; PD3: > 1000 parasites; AUC: *Area under the ROC curve; CI: confidence interval. ‘**–‘ *indicates values not shown due to non-significant p value.*

## Discussion

Significant advances in malaria control stem from improved diagnostics and treatments. RDTs targeting HRP2, LDH, and aldolase have revolutionized malaria detection with their speed and simplicity [22]. However, HRP2-based tests fail in regions with *pfhrp2* gene deletions, causing false negatives [6], while LDH and aldolase tests have reduced sensitivity at low parasitemia levels (10), necessitating alternative biomarkers. GDH and HDP, crucial for parasite metabolism and heme detoxification, respectively, show diagnostic promise due to broad *Plasmodium* species expression and functional conservation [23–25]. This study evaluates immune responses to GDH, HDP, and conventional markers (HRP2, LDH, and aldolase) under varying infection, endemicity, and parasitemia conditions to inform improved diagnostic strategies.

PCA of antibody responses demonstrated that the peptides effectively distinguished between malaria-slide negative and *P. falciparum*-positive samples. The clear separation between these groups highlights the robust discriminatory power of the evaluated peptides for infection status. However, PCA failed to reveal distinct clusters based on endemicity levels or parasitemia ranges, suggesting that these factors do not strongly influence the antibody profiles in this dataset. These findings suggest that while immune responses are sensitive to infection, they are less responsive to variations in malaria exposure intensity or parasitemia. This reduced responsiveness may be due to the plateauing of antibody levels following repeated exposure or chronic low-grade infections, which can blur immunological distinctions between individuals from areas of differing endemicity.

The Mann-Whitney U test confirmed significant differences in antibody levels between *P. falciparum-*positive and malaria-slide negative groups for all peptides (p < 0.005). However, comparisons across endemicity levels revealed that antibody responses to six peptides (GDH1, GDH3, HDP1, ALD1, ALD2, and HRP2) did not significantly differ between low and moderate endemicity. This pattern aligns with evidence that repeated malaria exposure induces sustained immune responses, reducing differentiation between closely related endemicity levels [26]. Interestingly, GDH2, HDP2, and LDH demonstrated significant potential in distinguishing between varying endemicity groups, providing valuable insights into their differential diagnostic capabilities.

Parasitemia classification also revealed specific distinctions. Antibody responses to GDH1 significantly differentiated between PD1 and PD3, while HRP2 distinguished between PD1 and PD2. These findings highlight the capacity of GDH1 and HRP2 to serve as parasitemia-dependent markers, contradicting earlier reports that suggested limited correlation between antigen load and immune response [27].

ROC analysis further supported the diagnostic utility of the peptides. All evaluated peptides exhibited AUC values exceeding 96% for distinguishing *P. falciparum*-positive from malaria-slide negative samples, confirming their robustness as reliable biomarkers for malaria detection. However, the AUC values for endemicity and parasitemia classifications did not exceed 75%, indicating reduced sensitivity and specificity for these categorizations.

When compared to current diagnostics, these peptide-based markers offer notable advantages. Microscopy, while species-specific and able to quantify parasitemia, is labor-intensive and operator dependent. RDTs, though widely accessible, are increasingly undermined by *pfhrp2* gene deletions and reduced performance at low parasitemia [6]. PCR-based methods provide high sensitivity but remain impractical for routine use due to infrastructure and cost constraints [28]. The peptides identified here demonstrated higher diagnostic accuracy under field-relevant conditions, positioning them as strong candidates for improved detection tools.

Remarkably, the antibody responses elicited by these peptides are well-suited for integration into Luminex-based multiplex assays, which enable simultaneous, high-throughput measurement of multiple immune markers with high sensitivity [29]. These platforms are ideal for sero-surveillance and large-scale screening, where cost-efficiency improves with scale and can support efforts to map transmission intensity, identify asymptomatic carriers, and monitor intervention impact [30].

This study provides a comprehensive immunological analysis of antibody responses to nine peptides in a large cohort of participants, offering valuable insights into their diagnostic potential for *P. falciparum* malaria. Our findings demonstrate that antibody levels against these peptides exhibit robust discriminatory performance, effectively differentiating *P. falciparum*-positive samples from malaria-slide negative samples. This is evidenced by PCA clustering, significant group differences, and AUC values exceeding 96%. These results highlight the potential for translating these findings into diagnostic tools that can accurately confirm infection with high sensitivity and specificity. However, there are several possibilities for future research to further validate and improve the clinical utility of these biomarkers. Future studies should focus on validating these biomarkers in diverse endemic regions, where varying transmission dynamics may influence their diagnostic performance. Additionally, integrating these biomarkers into multiplex RDTs could enable simultaneous detection of multiple antigens, improving diagnostic efficiency and reducing costs. Longitudinal studies assessing the utility of these biomarkers for tracking malaria exposure or reinfection over time could also provide valuable insights into their role in surveillance and monitoring of malaria control efforts. Expanding sample diversity, including individuals with mixed infections and non-malarial febrile illnesses, will be crucial in refining these biomarkers’ ability to distinguish malaria from other infectious diseases. A key limitation of this study is the inability to perform age-stratified analyses due to insufficient sample size within specific age groups, which may mask age-dependent variations in immune responses. Another limitation was using plasma instead of whole blood, which may affect field applicability. Plasma allowed controlled antibody quantification for early biomarker discovery, but whole blood is preferred for point-of-care use. Future work will optimize the assay for whole blood to enable rapid test deployment. The unequal sample sizes across transmission settings may have influenced comparative analyses, and the persistence of antibodies beyond the period of infection could result in false-positive findings. As diagnosis was based on microscopy, the possibility of undetected low-density or submicroscopic mixed infections cannot be fully excluded. Furthermore, the detection limit was not assessed using molecular methods, and the exclusion of submicroscopic and asymptomatic infections may limit the broader applicability of our findings for malaria elimination.

## Conclusion

This study suggests that immunological markers such as GDH and HDP hold promise for the detection of *P. falciparum* infections. The observed antibody responses indicate that these markers may help distinguish *P. falciparum*-positive samples from malaria-slide negative samples. While further validation in diverse populations and epidemiological settings is needed, the findings provide a foundation for exploring their integration into serological tools that could strengthen malaria surveillance and diagnosis in endemic regions.

## Supporting information

S1 Fig Principal component Analysis of antibody levels obtained against nine studied peptides among different endemicities (low, moderate, and High). (TIF)

S2 Fig Principal component Analysis of antibody levels obtained against nine studied peptides among different parasitemia groups including PD1(<200 parasites), PD2 (>200 to <1000 parasites) and PD3 (>1000 parasites). (TIF)
