# Supplementary figures and images for: Evaluation of GDH and HDP as novel serological biomarkers for Plasmodium falciparum malaria diagnosis across varying parasitemia and transmission settings in India
Source: PLoS One. 2025 Oct 15;20(10):e0334313. doi: 10.1371/journal.pone.0334313 (PMC12527120; doi:10.1371/journal.pone.0334313)

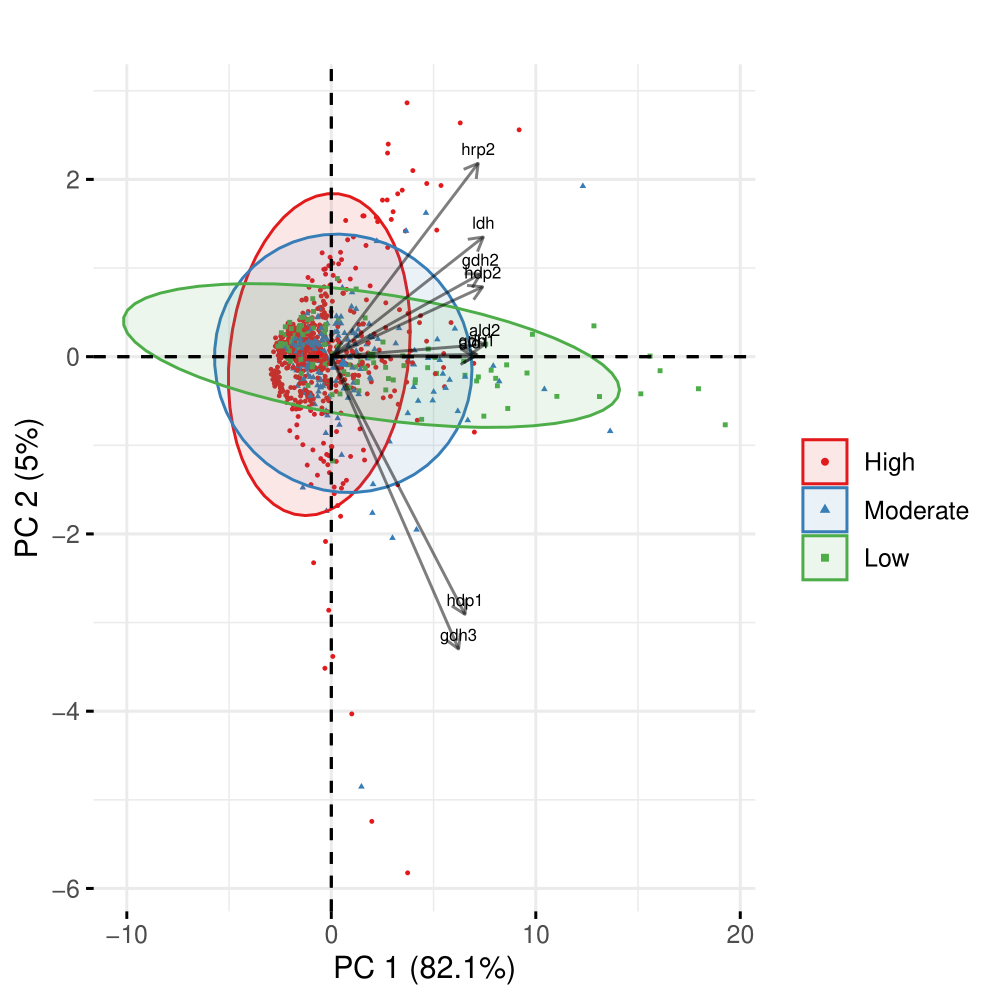

Supplement: S1 Fig — (TIF) [file pone.0334313.s001.tif]

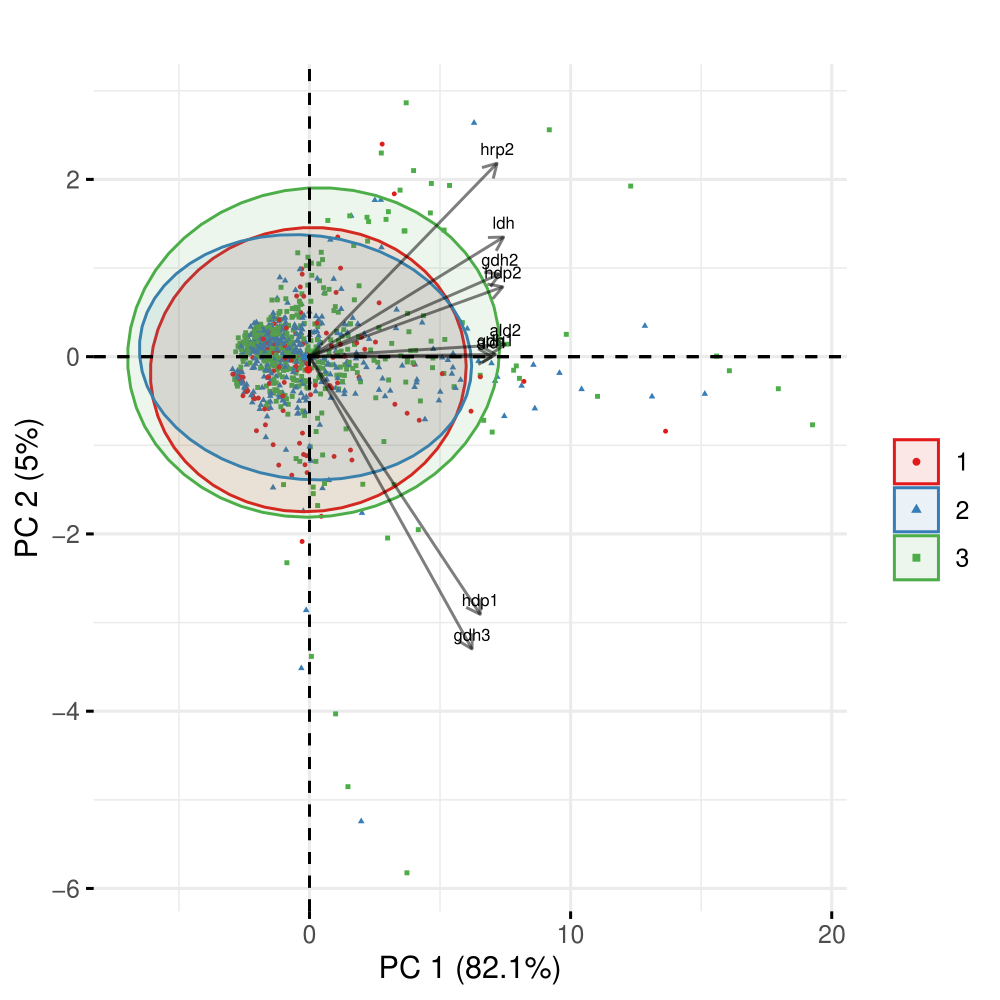

Supplement: S2 Fig — (TIF) [file pone.0334313.s002.tif]
